# Understanding the Thoughts and Preferences for Technologies Designed to Detect Feelings of Loneliness: Interview Study Among Older Adults

**DOI:** 10.2196/73694

**Published:** 2025-09-18

**Authors:** Jessica Rees, John Ratcliffe, Wei Liu, Yi Zhou, Sebastien Ourselin, Michela Antonelli, Ashay Patel, Yu Shi, Jingqi Liu, Anthea Tinker, Faith Matcham

**Affiliations:** 1 Institute of Gerontology, King's College London, London, United Kingdom; 2 School of Healthcare, University of Leeds, Leeds, United Kingdom; 3 Department of Engineering, King's College London, Strand Building, Strand Campus, London, WC2R 2LS, United Kingdom, +442078482747; 4 School of Biomedical Engineering & Imaging Sciences, King's College London, London, United Kingdom; 5 School of Design, University of Leeds, Leeds, United Kingdom; 6 School of Psychology, University of Sussex, Falmer, United Kingdom

**Keywords:** loneliness, older adults, preferences, qualitative, technology, mobile phone

## Abstract

**Background:**

Loneliness is a negative emotional state that is common in later life. The accumulative effects of loneliness have a significant impact on the physical and mental health of older adults. Automatic methods for detection and prediction are an emerging field to support early identification of loneliness.

**Objective:**

This study aimed to qualitatively explore the thoughts and preferences of people aged 65 years and older regarding technologies to detect feelings of loneliness in later life.

**Methods:**

We conducted 60 semistructured interviews with people aged 65 years and older between September 2022 and August 2023. Data were analyzed using a reflective thematic approach on NVivo software (Lumivero).

**Results:**

In total, three themes were identified representing what older adults considered important in a system able to detect loneliness: (1) interest and control of data, which was a priority for older adults; (2) perceived usefulness to address loneliness, which related to the importance of providing recommendations to reduce feelings of loneliness after detection; and (3) personalization as a priority, which included the level of loneliness for which an alert was sent and selection of relevant individuals who would be sent a loneliness alert.

**Conclusions:**

Findings from this in-depth qualitative study provide important perspectives from people with lived experience of loneliness on the context in which a sensor-based loneliness detection system would be most useful and acceptable to older adults. Future research will include such perspectives in the design of innovative technologies enabling the early detection of loneliness and access to timely interventions to tackle loneliness in later life.

## Introduction

Loneliness is widely acknowledged to be a serious public health matter [1]. Definitions of loneliness vary, but most authors agree that it refers to a negative emotion linked to poor quality and insufficient social relationships [2-4]. This is usually placed as distinct from social isolation, which refers to an “objective” lack of social relationships. The consequences of loneliness can include poor mental health [5], risk of Alzheimer disease [6], and increased mortality [7]. Aging has long been recognized as associated with loneliness [8], with later life even being expected to be a “time of loneliness” [9]. In the United Kingdom, as many as 1 in 4 people older than 65 years are experiencing moderate to high loneliness [10]. Age-related changes such as retirement, bereavement, reduced social networks, and loss of mobility are well-evidenced as risk factors for loneliness [8,11-14]. Older adults may experience transient loneliness, a temporary feeling experienced from time to time, during such age-related changes. If loneliness were to be felt consistently over a period of time, it would be defined as chronic loneliness, a state associated with worsening physical and mental health outcomes [15].

COVID-19 substantially impacted the feelings of loneliness and social isolation across the population, acting as a catalyst for many older adults to adopt the use of technologies in daily life [16]. For example, technology has been lauded for its potential to aid people in maintaining existing relationships and increasing communication with neighbors, family, and friends [17]. However, work has identified a multitude of barriers to uptake [18], including trust in the technology [19], and family caregiver concerns related to acting upon risks detected by technologies [20]. Issues of data protection, privacy, and control have been recorded in numerous studies looking at the use of digital technologies in health [21]. Automated technologies have the potential to challenge autonomy, particularly if they notify other people without the immediate consent of the older adult being monitored [22]. Despite such challenges, and with the acceleration of health care digitalization, wearable technologies for health monitoring present a timely opportunity to improve care for aging populations [23]. Due to the rapidly evolving digital landscape, the inclusion of older adults in the design of technology is imperative to avoid digital exclusion [24].

In recent years, using smart technology to identify loneliness has emerged as a potentially useful method of tackling loneliness in older adults [25]. Such technologies would monitor physiological or behavioral indicators of loneliness using sensors, enabling timely detection of loneliness to improve access to targeted intervention [26]. As loneliness is conceptually different from social isolation, which would detect the objective presence or absence of company, loneliness detection technologies are required to include several indicators, including stress, sleep, and activity levels, to enable the passive detection of such a subjective emotional state [27,28]. Specifically, sensor-based approaches measure the behavioral markers of social isolation, such as physical inactivity, poor sleep quality, eating behaviors, time out of home, and phone usage [29]. Numerous works have relayed instances of success using machine learning algorithms able to predict emotions using data such as galvanic skin response [30], electrocardiogram data [31], and electroencephalography data [32]. Behavioral data have also been applied to predicting loneliness [18,26,29]. For example, 1 scoping review found studies measuring daily life patterns to detect loneliness using “smart” technology (eg, ambient sensors to measure motion and touch), smartphones, and wearable sensors [19]. Another review of technology aimed at preventing loneliness in older people found that approaches measuring behavior and movement showed promise as they are usually successful at identifying loneliness [25].

The association between physiological markers of loneliness and the need to tackle loneliness in older populations suggests that it may be fruitful to construct technology capable of identifying loneliness in older adults [33]. Such technologies are required to be both useful and acceptable to older adults to be effective in supporting the reduction of feelings of loneliness. Relevant theoretical frameworks suggest that for technology to be accepted and used by older adults, it must be perceived as easy and useful, and users must have appropriate support and resources [34]. This study aims to gain insight into older adults’ general acceptance of loneliness detection technologies to identify the most meaningful priorities for this population, that are applicable to future technology development. Previous work has sought to understand older adults’ technology preferences and requirements [24], yet a gap remains in relation to older adults’ views on automated detection of loneliness specifically.

The potential to detect loneliness in older adults has numerous potential benefits. A wealth of evidence has suggested that loneliness may be a stigmatized condition [35]. Such negative desirability bias means the ability to detect loneliness without a person needing to seek help [36]. This may be particularly useful for older men who do not identify as lonely but suffer consequences of a similar nature [37]. Timely and accurate identification of loneliness in older adults will enable people to receive the right kind of support at the right time [26]. In general, approaches to alleviate loneliness in older adults include leisure activities (ie, gardening, music, and exercise), therapies (ie, animal, reminiscence, and cognitive), social and community interventions (ie, meal sharing), educational approaches (ie, relationship or skills training), befriending, or system-wide approaches [38]. Such activities can be made available to older adults through referral by primary care professionals through social prescribing initiatives [39].

This paper is part of the “Design for Healthy Aging (DELONELINESS)” project that aims to develop a smart monitoring and communication system with multifunctional electronics built into textiles used as wearables and home furniture to detect and measure loneliness levels in later life [26]. As outlined in previous publications, this system will involve the detection of physiological and behavioral indicators of loneliness in later life using sensor-based technologies. Such technologies will be worn by people aged 65 years and older, with data being inputted into an algorithm modeled to predict feelings of loneliness. Once loneliness is identified, the aim would be to enable users to receive support from family or health and social care professionals or promote engagement in activities to alleviate feelings of loneliness [26].

Including the perspectives of older people is central to the design of such a system [40]. In this paper, we present our qualitative findings on the thoughts and preferences of older adults on technologies designed to monitor and detect feelings of loneliness. Our specific objectives are to (1) describe the context and circumstances in which a smart system might be most useful; and (2) identify the most meaningful way of providing information back to individuals, their carers, or health care or social service providers.

## Methods

### Study Design

One-to-one semistructured interviews were conducted with participants aged 65 years and older on their experiences of loneliness and preferences for loneliness detection technologies. Detailed methodology used in this study is available in the published protocol [41].

### Ethical Considerations

Ethical approval was obtained from Research Ethics Committees at King’s College London (reference LRS/DP-21/22‐33376) and the University of Sussex (reference ER/FM409/3). Informed consent was sought from all participants. Participants were informed that they could stop their participation at any time. Participants were given a pseudonym following consent to ensure confidentiality. All identifiable information was kept in a password-protected Excel sheet on University OneDrive systems and removed at the point of transcription of interviews to protect the privacy of participants. Participants received a £30 (approximately US $40.47) LoveToShop voucher at the end of the interview as a thank you for sharing their experiences for the study. Interviews were audio-recorded and transcribed verbatim by a professional company.

### Recruitment

A stratified sampling technique based on age, gender, accommodation type, and level of digital ability was used. In terms of accommodation type, participants either owned their home, rented, or lived in sheltered accommodation, defined as housing for older adults consisting of private independent units with some shared facilities, emergency alert systems, and a warden [42]. We used the communication preferences of participants during the recruitment process (ie, if older adults had an email, mobile phone, or a landline number) as a proxy measure for the level of digital ability. Eligible participants were required to be aged 65 years and older, have experienced loneliness since reaching 65 years in age, be able to give informed consent, and speak English at a level fluent enough to take part in the interview. Study information was disseminated via research participant websites, organization newsletters related to housing, and through communication to individuals from previous research projects (related to depression and aging) who consented to being contacted about future studies. Interested individuals were invited to contact the research team to obtain study details. All participants provided written informed consent before data collection.

### Data Collection

Interviews were conducted between September 2022 and August 2023 by a female qualitative researcher and chartered psychologist with expertise in sensitive interviewing. In accordance with participant preferences, interviews were conducted either in-person (at home or in University offices) or remotely (via telephone or video call using Microsoft Teams software). To describe and stratify a range of experiences, we aimed to interview up to 60 individuals with lived experience in later life [41]. This number was chosen to enable the inclusion of a diverse range of participant characteristics related to loneliness, such as age, gender, accommodation type, level of digital ability, and health status. Characteristic data collected from participants included sociodemographic information (eg, age, gender, ethnicity, and accommodation type). We also included a measure of loneliness severity using the de Jong Gierveld Loneliness scale [43], which has 11 items related to social and emotional loneliness that are totaled to obtain an individual’s level of loneliness (ie, not lonely, moderately lonely, severely lonely, or very severely lonely).

The interview topic guide was developed by a multidisciplinary team of psychologists, gerontologists, product designers, and machine learning experts. The first part of the interview focused on the subjective experiences of participants to identify the signs and associated behaviors with loneliness in later life. Findings from this section of the interview have been previously published [33]. The latter half of the interview focused on participants’ thoughts and preferences on the role of technology to detect loneliness. In line with theoretical models for technology acceptance [44], the interview topic guide contained specific questions related to older adults’ perceptions of the usefulness of technologies. Refer to Multimedia Appendix 1 for complete details.

In relation to technology, we asked about participants’ existing use of technologies in relation to their health. We then introduced proposed technologies for measuring loneliness, for example, sensors worn on the body or integrated into a fabric which collect physiological data that may be relevant to loneliness (ie, heart rate and activity levels). We asked participants where on their body or in their home they would be willing to have a device. Finally, we asked about data collected by loneliness detection technologies. Specifically, we asked how useful they would find data collected by the system (ie, sleep, movement, and stress levels) and if these data could be sent to anyone to improve participants’ quality of life. We then asked about the benefits and limitations of the data to participants, family members, and health care professionals. Finally, we asked about participants’ thoughts on receiving an alert to the risk of becoming lonely, and what participants would hope family members or health care professionals would do if they received a loneliness alert. Refer to Multimedia Appendix 1 for the full interview schedule.

### Analysis

Data were analyzed using a reflexive thematic approach by the lead author (J Rees) using NVivo software (version 14; Lumivero) [45]. The early structured themes were constructed by identifying “semantic” themes – surface-level ideas built from the stated perspectives of the participants, but which are not thoroughly interrogated [46]. The generation of initial codes was generally grouped in line with the topic guide questions, for example, usefulness of data, benefits and limitations to data, and thoughts on loneliness alert. We used NVivo to organize our themes into the perspectives of “older adults,” “family members,” or “health care professionals.” These were transformed into “reflexive” themes through several intertwined processes. Specifically, we wrote up “semantic” themes fully into a report, which was then shared with the multidisciplinary project team and discussed in team meetings. Such discussions lead to the refinement of themes, specifically distinctions between theme definitions. Themes were reviewed a final time by the second author (J Ratcliffe), who was involved in drafting this paper but was not present during data collection and analysis. To facilitate an iterative process of theme building and refinement, the themes were critically contrasted with extant literature [47], specifically the literature on interventions for reducing loneliness. In addition, to extrapolate information on “what works for whom in which contexts” [48], participant characteristics, specifically level of loneliness, marital status, and accommodation type, were considered throughout the write-up stage.

## Results

### Participant Characteristics

In total, 60 older adults were interviewed with a mean age of 73 (SD 6.7) years, ranging from 65 to 91 years. Most participants were aged between 65-74 years, women, White British, homeowners, and with a moderate level of loneliness (Table 1).

**Table 1.** Participant demographic data (N=60).

Characteristics	Participants (N=60), n (%)
Age (y)	
65‐74	42 (70)
75+	18 (30)
Gender	
Women	42 (70)
Men	18 (30)
Ethnicity	
White or White British	54 (90)
Black or Black British	3 (5)
Asian or Asian British	3 (5)
De-Jong-Gierveld scale loneliness level	
Not lonely	3 (5)
Moderate	36 (60)
Severe	10 (17)
Very severe	11 (18)
Accommodation type	
Own home	43 (72)
Rented	10 (17)
Sheltered accommodation	7 (11)

Marital status varied from widowed (n=20), divorced (n=16), single (n=11), married (n=8), and separated (n=5). The frequency of loneliness also varied from often or always (n=23), occasionally (n=16), some of the time (n=20), and never (n=1). Most participants were degree-educated (n=34). In total, 30% (n=18) of participants did not have children, and 13% (n=8) did not have anyone to contact if they were in trouble or needed help. The majority of participants reported having a long-standing illness or disability (n=50), such as depression, chronic fatigue, arthritis, or hearing loss. Furthermore, 5 participants communicated using their landline numbers only.

### Themes

#### Summary of Themes

In total, 3 themes were constructed to represent older adults’ thoughts and concerns regarding a loneliness detection device. First, “interest and control of data” outlined the characteristics of older adults interested in a loneliness detection system and the importance of retaining control over data collected to detect loneliness. Second, “perceived usefulness to address loneliness” highlighted the need to provide suggestions to reduce loneliness in conjunction with an alert about loneliness. Third, “personalization as a priority” relays how participants felt the system needed to be tailored to individuals, particularly regarding (1) the level of loneliness that required intervention, and (2) who the participants felt should be informed they are lonely. Refer to Table 2 for theme outline including supporting quotes.

**Table 2.** Themes and selected supporting quotes for thoughts and preferences of older adults for technologies designed to detect feelings of loneliness.

Theme	Supporting quotes
Interest and control of data	“So it’s, well it’s just, you know, curiosity about how your body is working. And trying to find patterns and things which is obviously what this is trying to do anyway.” [P18, Female, 70, Married, Very Severe] “Well it’s simply because I know I would only get annoyed because I know I’m not doing many steps. Although it’s interesting too the way it does your heart rate. Now that’s something again I would use it.” [P15, Female, 66, Divorced, Moderate] “Can’t be bothered with things like that..I’m not going to make a ridiculous effort at carrying technology around to count them because it’s kind of not relevant.” [P52, Female, 66, Widow, Not Lonely] “That’s the barrier to me. The information, if it’s kept simple, if it’s something that’s user-friendly to me that’s not terribly keen to embrace actively too much technology.” [P35, Female, Married, Moderate] “Yes, some things I might know already but it might show some others that I was not already aware of and that is nice you know, it might not be pleasant, but it is happening and that is good.” [P23, Male, 68, Separated, Not Lonely] “The technology is there. I’d like it all to be used for good but I’ve no control over whether it is or it isn’t and I think for my lifestyle and stuff if you’ve got nothing to hide it doesn’t really matter.” [P13, Female, 69, Separated, Moderate] “I’d be a bit- your terminology, ‘at risk of becoming lonely,’ that’s- a bit kind of- big brotherish, really. I suppose- perhaps it’s just the wording, if you said, oh we’re concerned for you, it’s about time you got out a bit more, then- go and find some friends, go out and have a sing song or go out for a run.” [P47, Female, 72, Widowed, Severe]
Perceived usefulness to address loneliness	“I don’t think it would be any use to me. because I know how I feel. I know how I felt yesterday, but I’m feeling better today. No, I don’t think it would bother me. I’m not a great one for data. It’s more likely to be useful to someone else who’s trying to find patterns or (I don’t know) reasons for being low.” [P8, Female, 65, Married, Moderate] “I don’t know that I could do anything about it, so what’s the point in telling you, I don’t know. I mean it’s because they’re going to have someone phoning you up and having a chat with you, you know a silver line chat sort of thing. I don’t know if that would make me feel less lonely I suppose you might think well someone could be bothered.” [P9, Female, 72, Divorced, Very severe] “Yes, to have the results but also to have the course of how to break that. It’s no point telling me I’m lonely which I already know right. A flashing thing saying please, do this, that might be helpful because there is more directive than giving information that somebody is lonely which they most probably know already.” [P49, Female, 72, Divorced, Severe] “If the algorithms or whatever they call them, if it sees that predicted pattern and it happened once before it says, Oh, last time when you were in that- what actually brought those levels down was that you went and did this.” [P35, Female, 68, Married, Moderate] “For me would be an additional motivator because I’ve got some concrete evidence, so it would be a motivator to say, Look you’re feeling lonely now is the time to make that effort to walk up the pub because there’s some music going on tonight, and you know you’re going to see so and so.” [P32, Male, 70, Widowed, Moderate] “I don’t feel I would need that at this moment in time. I don’t but we can’t see ahead can we, into the future, and if, when I was, if I was much, much older than I am now then I would probably think it would be a good thing to have but at this moment in time I’m not there.” [P55, Female, 73, Single, Moderate]
Personalization as a priority	“And you could choose the point at which it could be shared, if you like, with those people so that, yes, don’t bother to tell them if I’m feeling lonely 10% of the time, but maybe if I’m feeling lonely 50% of the time, then do. But it might be different for other people.” [P52, Female, 66, Widowed, Not lonely] “I think it would be good, because I don’t get as much time with my GP as I’d like. I think if she was receiving this information and give her something to study about me, so when I did see her, she could refer back and say, Oh I’ve noticed your sleep isn’t so good. I’ve noticed you’ve had these waking moments. But I see that your heart rate’s good. So that must be good for you, you know?” [P5, Female, 65, Divorced, Severe] “When you say you’d alert somebody, who would you alert in my case? I’ve got no one close that you could send an “I need help” message to.” [P43, Male, 70, Widowed, Moderate] “I really wouldn’t want her to be worrying about me, she has her own problems, I think that’s something I can cope with. If it’s only feeling a bit lonely, I can cope with that, and I really don’t think it’s worth worrying somebody else. I mean, what are they going to do? Get out of work and rush over just to sit in the house and talk to you, you know I don’t know.” [P25, Male, 68, Divorced, Very Severe]

#### Theme One: Interest and Control of Data

Older adults spoke about how health data collected by the system (ie, heart rate, sleep, and activity levels) were interesting, comforting, reassuring, and empowering (“knowledge is power”). These participants all had previous positive experience with using smartwatches. However, some participants were not interested in these data either because they had no present health issues and did not wish to worry about their health (“it is not something you can do anything about anyway”) or because of their general disinterest in the use of technology (“I am not a great one for data”).

Older adults had varied technological ability, with some using virtual reality and online shopping, whereas others had no WiFi or a sense that there is “too much” technology in daily life. These categories were not mutually exclusive. For example, the oldest participant in the sample (aged 91 y) used technology on a daily basis, including a smartwatch or phone, but felt it isolated her further by reducing in-person interactions, which was especially impactful as she lived in a rural area. Similarly, some participants expressed worry about who would access the data and felt being monitored closely would be intrusive, yet others felt comfortable with data, describing themselves as “open minded” with “nothing to hide.” Such perspectives were not associated with the age of the participants or the severity of loneliness.

*I imagine one of your questions is going to be about the use of data and stuff like that. I’m very comfortable with that…I don’t think Big Brother’s going to have a huge interest in how many times my heart beats.*

[P1, Female, 68, Widowed, Moderate]

The main preference described by older adults was the need to be in control of both the data collected and an alert sent by the system. It was important for older adults to understand which data were being collected and for what purpose. Older adults highlighted how it was important to be aware of the behaviors associated with loneliness and to know why they were being alerted.

*If the algorithms or whatever they call them, if it sees that predicted pattern and it happened once before it says, Oh, last time when you were in that- what actually brought those levels down was that you went and did this.*

[P35, Female, 68, Married, Moderate]

It was important for older adults to feel in control of their data. Participants wished for any data or alerts to be sent to them directly and have the option of contacting other people if they deemed it necessary (“I’d much rather it came through me”).

*The less control you have, the more control you need. So, people will control what they can control. And therefore, it’s important that the end user has the control over how things are shared.*

[P52, Female, 66, Widowed, Not Lonely]

#### Theme Two: Perceived Usefulness to Address Loneliness

Most participants described already being aware of their loneliness and did not see the benefit in being alerted about loneliness by the system (“I know I’m lonely I don’t need an alert”). Linked with the severity of loneliness, some older adults felt that if you did not notice feelings of loneliness, then a person was not truly feeling lonely.

*You’re aware of the build up. If you aren’t aware of it, then you’re not of that degree of loneliness, you’re just feeling low, can I put it that way? Whereas we can all feel low, and have a bad half hour, a bad day, but loneliness is something else that is different.*

[P40, Female, 75, Single, Moderate]

Nevertheless, some thought that the system would be a useful prompt as it provided “concrete evidence” to the early warning signs of loneliness and would either “consolidate” their recognition of loneliness or motivate older adults to engage in behaviors to reduce loneliness. Older adults highlighted how it was important to be aware of the behaviors associated with loneliness and to know why they were being alerted. One participant compared the system with a “companion” for people who felt alone, whereas others felt the system would have been useful in the past (eg, during widowhood and retirement) or in the future (eg, if cognitive ability reduced). The majority of participants (n=45) lived alone, and this group in particular expressed a concern about falling in the home and suggested that the loneliness sensors could also be used to detect this.

Older adults questioned the use of the system if there was no consequence or follow-up to loneliness detection. Participants felt that if they were to be alerted of loneliness, there would also have to be a recommendation to reduce feelings of loneliness. This requirement was consistently expressed across participants, irrespective of age, gender, marital status, or living situation.

*If you could tell me how to reduce it, what to do, that would be more important than letting me or anybody else know that I was feeling- I know that I’m feeling it or about to feel it. But it’s what to do about it that’s the critical thing…There’s no point in it otherwise.*

[P41, Female, 84, Divorced, Severe]

#### Theme Three: Personalization as a Priority

##### Subtheme 3a: Level of Loneliness

Older adults found it important to decide on a level of loneliness at which the alert would be sent to themselves. As participants had a variety of loneliness experiences, this level would need to be tailored to individuals. For example, widowed older adults experienced transient loneliness (ie, more severely at certain times), which may be time-limited, depending on individual grief trajectories. Some older adults experienced severe loneliness, where they were more likely to describe chronic loneliness and thus questioned which level of loneliness would be most appropriate for them. One male participant, who experienced chronic loneliness despite living in shared accommodation, felt loneliness at a consistent level at all times, so he questioned what changes the system would be able to detect.

*You’ve got to decide on a level of stress that, you know, like we do on a scale of 1 to 10. You know, if I feel 3 amount of lonely, then don’t bother, it will probably pass over, but if I feel 7 or 8 amount of loneliness then please tell me to get off my bum and go out for a walk or something.*

[P47, Female, 72, Widowed, Severe]

Participants varied in their responses describing how they would engage in activities to alleviate loneliness following an alert. Some participants described preferring to manage feelings of loneliness on their own, especially for low levels of loneliness (“if it’s only a bit lonely I can cope”). When discussing potential interventions following a loneliness alert, participants highlighted the importance of personalization as “every person is going to be different.”

##### Subtheme 3b: Involvement of Others

Participants also discussed the importance of personalization in relation to the involvement of others, specifically health care professionals and family members. For example, some participants had previously experienced suicidal thoughts during times of loneliness and felt it was important for health care professionals to be alerted if they experienced loneliness at that level in the future. However, many questioned whether busy health care professionals, such as general practitioners, had the resources to help with loneliness.

*…the GP, he can’t give me a tablet to stop me being lonely or something like that. Can’t come round and spend time with me, you know. They haven’t got time to see you in the surgery. So, yes I mean I wouldn’t mind the GP knowing I was lonely. But I can’t see what advantage there would be there.*

[P46, Female, 87, Widowed, Moderate]

Nevertheless, some felt the alert could be sent to health care professionals for severe loneliness or where loneliness was related to physical or mental health issues, such as depression, and some mentioned signposting to counseling and social-prescribing services.

Similarly, older adults felt that loneliness would have to be “severe enough” for the system to contact family members and that this level of severity would differ between individuals.

*And you could choose the point at which it could be shared, if you like, with those people so that, yes, don’t bother to tell them if I’m feeling lonely 10% of the time, but maybe if I’m feeling lonely 50% of the time, then do. But it might be different for other people.*

[P52, Female, 66, Widowed, Not lonely]

When participants experienced mild feelings of loneliness, many did not want friends or family members to send the loneliness alert, stating they would feel “embarrassed” or “guilty,” and that they could cope with it alone to avoid being a “burden.” Some participants expressed not having a problem with family receiving a loneliness alert; however, they questioned what they would be able to do about it if they lived far away. In more severe cases of loneliness, participants thought the loneliness alert could be used as a “conversation starter” during lonely periods where older adults felt less able to initiate social contact. However, others still felt the alert was “too private” to share with others without previous consent from the older adults themselves. Vitally, other participants had no family, so they questioned who they could nominate to receive a loneliness alert. This was especially pertinent for single participants over 80 who felt the alert would be an additional burden to next of kin and relied on safety alert systems in sheltered accommodation in an emergency.

*When you say you’d alert somebody, who would you alert in my case? I’ve got no one close that you could send an “I need help” message to.*

[P43, Male, 70, Widowed, Moderate]

Overall, involvement of family, friends, and health care professionals in a smart system to detect loneliness was thought to be part of a negotiation as to who the older adult would want involved, at what time, and for what purpose.

## Discussion

### Principal Results

We constructed 3 themes to represent what older adults considered important in a system able to detect loneliness. “Interest and control of data” highlighted general interest in health data being a personal preference of older adults based on current or previous positive use of technologies. Participants had varying technological abilities but were unanimous in their need to be in control of the data collected and sent by the system. “Perceived usefulness to address loneliness” refers to whether and how the system can be useful to older adults. Many older adults described being aware of feelings of loneliness, especially in severe cases, and thus questioned the usefulness of the loneliness detection system. Participants emphasized the importance of providing recommendations to reduce loneliness following detection to improve usefulness. Finally, “personalization as a priority” refers to the need to work with the individual using the system to determine the level of loneliness that results in an alert, and the consequences of that alert, including the involvement of others to support loneliness, such as friends, family members, or health and social care professionals.

The focus on control echoes concerns over privacy and data protection reported in previous studies on technology use in later life [22,49,50]. Loneliness has been associated with a lack of autonomy [51]; therefore, it is vital that older adults feel in control of the data collected and sent by a loneliness detection system. Trust in technologies can be established through providing feedback in real time and through extensive validation [17,19]. Technology-based approaches for reducing social isolation may not be appropriate for all older adults [52]. As a “digital divide” exists between those with cultural and financial access to technology and those without [24], research has found that lonelier older adults use information and communication technologies less often than their less lonely counterparts [53]. Factors that were found to influence engagement include lack of skills and support to use digital health technologies [54] and a fear or dislike of technology [21]. Ensuring that older adults across a spectrum of abilities feel knowledgeable and confident with the data and associated alerts from the loneliness detection system, is therefore paramount to its use.

The findings of this study suggest a key component of ensuring acceptability of a loneliness detection system is the effective implementation of personalization. Our findings support previous literature, which suggests that digital health systems should make it transparent as to who will gain access to the information collected, and ensure users retain control of who has access to it [55]. A novel finding from our work is the importance of determining an individually selected level of loneliness for which an alert would be sent, highlighting the subjective nature of loneliness [15]. Further consideration is required as to how degrees of loneliness would be measured and reported to the system, for example, self-reporting from older adults or the use of validated loneliness scales [56]. More broadly, our findings on personalization indicate the importance of “user-centered” design in health technology (perspectives that centralize the needs of the user in the design and implementation of the technology) [29,57,58]. This is especially important in the context of culture, which plays an important role in shaping an individual’s experience of loneliness [59]. Developing culturally sensitive interventions to reduce feelings of loneliness is an important priority for future research to ensure targeted support is available for older adults.

In relation to the involvement of others in supporting loneliness once detected, older adults emphasized that whoever the system alerted, this should be decided in advance via a clear and transparent set of discussions. Participants tended to refer to general practitioners when health professionals were discussed, a finding that may mirror the trust instilled in doctors by older people [60,61]. Previous research on technology preferences of older adults has identified that if a device was recommended to older adults by a “credible” health professional, it was more likely to be taken up and used [24]. The participants in this study generally held negative attitudes toward alerting family members of loneliness, mirroring concerns from previous research [62]. However, friends or family were not consulted in this study; therefore, it is possible that their thoughts on receiving an alert for loneliness may not align with those of older adults. Perceptions of burden were described as one reason for participant disapproval of family member involvement. Indeed, loneliness has often been linked with guilt, shame, and a perception that others do not care [3,63]. Further research is required on the involvement of family or friends in providing support following an alert from a loneliness detection system, as many loneliness reduction interventions actively involve others [64].

Perceived usefulness is a core component of theoretical models of technology adoption in older adults [44,65]. Participants had varied perspectives on whether and how a loneliness detection system could be useful. Some saw it as potentially useful as a prompt to change their own actions or as an “early-warning” sign before they would have thought to seek help, perhaps even before they realized that they were lonely. Given the stigma of loneliness [3] and the reticence of older men to identify as lonely [63], such a system may be particularly useful for removing the anxiety and uncertainty of doing something about loneliness. Older adults in our study were clear that recommendations to reduce feelings of loneliness once detected were an imperative aspect of such a system. Research has highlighted how there is “no one size fits all” approach to loneliness intervention and that tailored, targeted approaches are required to reduce loneliness (Victor et al [66]). Detailed consideration is required on the selection of appropriate interventions for older adults using a loneliness detection system based on the type of loneliness (ie, emotional or social), severity of loneliness (ie, moderate, severe, or very severe), and reason for loneliness (ie, bereavement or mobility issues). Future research will be required on the acceptability and effectiveness of such recommendations, noting that individual-centered interventions are inherently limited by their lack of capacity to influence the social context of the lonely individual [3].

### Limitations

This study adds to the emerging literature on sensor-based technologies to detect feelings of loneliness in older adults [18,29]. Our large sample size enabled us to present a variety of perspectives and incorporate a diverse range of participant characteristics, including those who were skeptical or ambivalent about the technology. Despite efforts to achieve an equal distribution of gendered and cultural perspectives, our sample was majority female White-British. Our themes may therefore not represent the views of older adults with different perspectives on technologies, which requires further consideration in future design testing. Our interview sample included people with lived experience of loneliness to provide relevant perspectives and expertise to inform future technology development [67]. It is important to acknowledge that such participants were more open to acknowledging and seeking help for loneliness than some of the prospective target population, who may not readily seek help for loneliness. Social desirability bias may have also influenced how participants spoke about technology. Thus, a gap exists in our knowledge of older adults with reduced help-seeking behavior that may particularly benefit the use of a loneliness detection system. Finally, the perspectives of participants were based on a hypothetical loneliness detection device described by the lead author conducting the interviews. There is no such actual device yet; therefore, potential barriers and enablers identified in related research, such as comfort, cost, and maintenance [68,69], could not be effectively discussed or reviewed. Interpretations of the loneliness detection system may vary among participants, and further user testing would be required to obtain information on acceptability once a device to detect physiological markers associated with loneliness has been developed [33].

### Conclusion

Findings from this large qualitative study provide important perspectives from people with lived experience of loneliness on the context in which a sensor-based loneliness detection system would be most useful and acceptable to older adults. It is important to support older adults across a spectrum of abilities and preferences to feel knowledgeable and confident with technologies used by a future loneliness detection system. For older adults to feel in control, transparency on data collected by the system is required in addition to options for personalization related to levels of loneliness, which creates an alert, and which supporters are informed. This should be identified through a clear and full discussion, before they commence using it, of when, why, and who the device should alert in the event of it detecting loneliness. The perceived usefulness of a future loneliness detection system is reliant on activities to reduce feelings of loneliness being recommended following the identification of loneliness. Future research will include such perspectives in the design of innovative technologies enabling the early detection of loneliness and access to timely interventions to tackle loneliness in later life.

## Supplementary material

10.2196/73694 Multimedia Appendix 1 Topic guide and semistructured interview.
