# Supplementary material for: Understanding the Thoughts and Preferences for Technologies Designed to Detect Feelings of Loneliness: Interview Study Among Older Adults
Source: JMIR Hum Factors. 2025 Sep 18;12:e73694. doi: 10.2196/73694 (PMC12445782; doi:10.2196/73694)
Supplement: Multimedia Appendix 1 [file humanfactors-v12-e73694-s001.docx]

# **Multimedia Appendix 1. Topic guide**


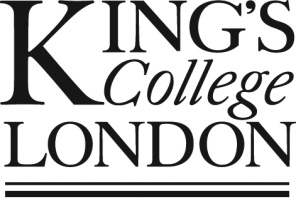
**SEMI-STRUCTURED INTERVIEW**

*Ethical Clearance Reference Number:* LRS/DP-21/22-33376

**Exploring the Psychological Experience of Loneliness**

The primary purpose of this interview is to gain insight into the loneliness in older people, and to inform sensor development work being conducted within the DELONELINESS project. The purpose of this interview is threefold:

1. identify the psychological and social parameters of loneliness, prioritized for consideration within a smart system;
2. describe the context and circumstances in which a smart system might be most useful; and
3. identify the most meaningful way of providing information back to individuals, their carers, or healthcare/social service providers.

Anonymized quotes may be used in internal reports, external publicity (such as soundbites on the DELONLINESS website), and for research purposes. The interview will be a maximum of 2 hours, and will be recorded for future reference. This guide is designed to provide a structure for interviewers to follow, but does not rule out opportunities to adapt or change the questions, or their order, depending on what the interviewee says.

| **Interview Phase/Purpose** | | **Questions/Prompts** |
| --- | --- | --- |
| **SECTION 1: Experiences of loneliness** | | |
| **Definition of loneliness** | 1. Can you tell me how you describe loneliness? What does the word mean to you? 2. Do you identify with another word? (social connection, social isolation, aloneness, solitude).   *Prompt*:   - Social loneliness (discrepancy between actual and desired quantity and quality of social interactions, includes cultural differences). - Emotional loneliness (absence of meaningful relationships, negative feels can occur ever in close contact with people). - Existential loneliness (sense of separateness from others and wider world, particularly being illness and bereavement). - Relationship-specific loneliness (romantic partner, siblings, children, friends, community). | |
| **Confirming experience of loneliness**   - Identify when the individual felt lonely | | You mentioned in the questionnaire that [insert answer from loneliness measures]. Thinking about these aspects can you tell me:   1. Please describe the last time you felt lonely? 2. How long did you feel lonely for?   *Prompts: Did you feel lonely for weeks or months or years? Or for hours and days? Different before/after COVID?*   1. How long ago were you feeling this way, or are you still feeling lonely? 2. Did you feel lonely all the time or was it broken up by periods of not feeling lonely? |
| **Personal experiences of loneliness** | | 1. When you think about the most significant period of loneliness you’ve experienced since your 65^th^ birthday, can you describe how it felt?   *Prompts: Describe anything that comes to mind – your mental health, social life, relationships, physical health…* |
| **Correlates of loneliness**   - Establish the holistic experience of loneliness. | | “Thank you for sharing that with me. You mentioned that [insert answer to Q7] was associated with the loneliness you experienced. Can you tell me more about that?  *Prompt: Did they occur at the same time? Can you describe the experience in more detail? Did X finish when the period of loneliness had ended?*  Repeat for all the things listed for Q7… |
| **Precursors to loneliness**   - Determine what things might be able to predict loneliness. | | Thinking about the most significant period of loneliness you’ve experienced since turning 65…   1. Can you remember anything happened in the weeks, months or years leading up to it which might have contributed to it?   *Prompts: Had you had any changes to your usual routine, life events, or diagnoses? COVID-19?*  “You’ve mentioned that [insert answer to Q8] happened shortly before your most significant period of loneliness.”   1. How long, roughly, before you started feeling lonely, did you start to experience this? 2. Is there anything that could have stopped [insert answer to Q8] from influencing your loneliness? 3. Is there anything else you haven’t mentioned so far that could have been associated with the onset of your most significant experience of loneliness?   *Prompts: Feel free to mention anything, no matter how big or small it might have seemed at the time.* |
| **Implications of loneliness and support received**   - Understand what the end of a loneliness event might look like and the care pathways used. | | 1. Thinking about your most significant period of loneliness, can you tell me more about the aspects of your life it affected?   *Prompts: Describe anything that comes to mind – your mental health, social life, relationships, physical health…*   1. How did the loneliness end? Did it finish naturally, or was there an event or intervention which helped it go away? 2. If something specific helped it go away, can you explain what happened?   *Prompts: Did you actively seek help? Who did you speak to? What did they do? If you didn’t actively seek help, what triggered the change in your loneliness.*   1. If your loneliness is ongoing, what do you think would help it go away? |
| **Final thoughts**   - Opportunity to discuss anything else | | “Is there anything else about your experience of loneliness which you would like to share before we move onto the next part of the interview?” |
| **OPPORTUNITY FOR A BREAK IF REQUIRED** | | |
| **SECTION 2: The role of technology in measuring loneliness**  “Thank you for sharing such personal information about your experiences of loneliness. The next part of the interview is going to move on to talk about who technology might be used to help us measure loneliness more effectively.” | | |
| **Environment and daily routine**  Establishing patterns of activity when lonely and when not lonely | | “I would like to move on by asking some questions about your daily routine and your living environment.”   1. Describe your living environment to us please? 2. Which room/furniture in your living environment do you use the most? How long?   *Prompts: Are you living in a flat or a house? How many rooms are there? Do you have regular house guests?*   1. Can you describe a typical daily routine for you?   *Prompts: What time do you tend to wake up? Do you have the same breakfast every day? Do you leave the house every day?*   1. Does this routine change much when you’re experiencing loneliness? 2. [If yes] What tends to change in your daily routine when you’re lonely? |
| **Existing use of technologies** | | 1. Do you use technology to measure any aspect of your health right now?   *Prompts: This could be an app on your phone, a website you access regularly, or some kind of technology that you wear or carry with you to measure something.*   1. [If yes], please tell me more about it.   *Prompts: How often do you use it? What works well/favourite? What works less well/least favourite?* |
| **Data collection requirements**   - Identify priorities for the smart system | | “Part of the [DETAILS REMOVED FOR ANONYMISATION] study is developing new ways of measuring loneliness, through sensors which might be worn on the body, or integrated into a fabric. These sensors will measure things, such as heart rate, or small movements you make, which might be a useful indicator of loneliness.”   1. Thinking now about a device which you might wear on your body, where on your body would you be willing to wear a device?   *Prompts: Would you be willing to wear something around your wrist like a watch? Would you prefer something on your ankle, or attached to your chest?*   1. Wrist: what would make you less willing to wear this device every day? What would make you more likely to wear this device every day? 2. Would you be willing to wear the device all day, or would you want to be able to take it off sometimes? Why would you want to take it off?   Another option might be to have a sensor which is in fabric in your clothes, or in furniture.   1. If we were to integrate the sensor into fabric in a piece of furniture, what would be the most convenient piece of furniture to use? 2. What would make you less willing to use this piece of furniture every day? What would make you more likely to use this piece of furniture every day? 3. If we were to integrate the sensor into fabric in an item of clothing, what type of clothing would be most useful? 4. What would make you less willing to wear this item of clothing every day? What would make you more likely to wear this item of clothing every day? 5. Would you rather have clothing which had a sensor in, or would you rather have a piece of furniture with the sensor in? 6. What types of materials or fabric bring you the most comfort at home? |
| **Loneliness impact on engagement**   - Establish whether answer to any of these questions might be different when lonely. | | Thinking about the conversation we’ve just had about your preferences for wearable technologies and textile sensors.   1. Do you think any of your opinions or requirements would change if you were feeling lonely? 2. If you’re currently feeling lonely, do you think any of your opinions or requirements would change if you weren’t feeling lonely right now? |
| **Final thoughts**   - Opportunity to discuss anything else | | 1. Is there anything else about your preferences or requirements for a device which you haven’t had an opportunity to mention so far? |
| **OPPORTUNITY FOR A BREAK IF REQUIRED** | | |
| **SECTION 3: Data feedback and integration into services**  “Thank you for giving us an insight into your living environment and your preferences for how we could be measuring loneliness using sensors. We’re now going to move onto the final part of the interview, which focuses on what we should do with the data we collect. We might be able to collect data about a wide range of things, such as your sleep, movements and stress levels.” | | |
| **Data recipients**   - Who should receive it? | | 1. How useful would this data be for you, your family or caregivers and your GP to receive? 2. Is there any other person or service this data could be sent to help improve your quality of life? |
| **Implications of data**   - What the data would mean | | 1. If this data were sent to you…what benefits are there to having this kind of information available?   *Prompts: Might this data change your daily routines, or prompt you to do something different?*   1. What limitations might there be to having this kind of information available?   [repeat questions for family or caregiver and GP if relevant] |
| **Data requirements and actions**   - How data might be received | | 1. What would you do if we were to alert you that you were at risk of becoming lonely? 2. If this data were sent to you…how would you want to receive it? How often? 3. What would you hope a carer or family member/GP would do if we were able to alert them that you were at risk of becoming lonely? |
| **Final thoughts**   - Opportunity to discuss anything else | | 1. Is there anything else about your what we could do with the information we collect you haven’t had an opportunity to mention so far? |
